# A novel approach to improve hand hygiene compliance of student nurses

**DOI:** 10.1186/2047-2994-2-16

**Published:** 2013-05-30

**Authors:** Sharon Salmon, Xiao Bei Wang, Theresa Seetoh, Siu Yin Lee, Dale A Fisher

**Affiliations:** 1 Infection Control Team, National University Health System, 1E Kent Ridge Rd, Singapore 119228, Singapore; 2 Nursing Administration, National University Health System, 1E Kent Ridge Rd, Singapore 119228, Singapore; 3 Nursing Education Unit National University Health System, 1E Kent Ridge Rd, Singapore 119228, Singapore; 4 Ministry of Health Holdings, 1 Maritime Square, #11-25 Harbour Front Centre, Singapore 099253, Singapore; 5 Department of Medicine, National University Health System, 1E Kent Ridge Road, NUHS Tower Block, Level 10, Singapore 119228, Singapore; 6 Department of Medicine, Yong Loo Lin School of Medicine, National University of Singapore, 5 Lower Kent Ridge Road, Singapore 119074, Singapore

**Keywords:** Compliance, Hand hygiene, Infection control, Nursing students, Nursing education

## Abstract

**Background:**

The National University Hospital, Singapore routinely undertakes standardized Hand Hygiene auditing with results produced by ward and by staff type. In 2010 concern was raised over consistently low compliance by nursing students averaging 45% (95% CI 42%–48%) prompting us to explore novel approaches to educating our next generation of nurses to improve their hand hygiene practice.

We introduced an experiential learning assignment to final year student nurses on attachment to NUH inclusive of hand hygiene auditor training followed by a period of hand hygiene observation. The training was based on the World Health Organisation (WHO) “My 5 moments for hand hygiene” approach. Upon completion students completed an anonymous questionnaire to evaluate their learning experience.

**Findings:**

By 2012, nursing students were 40% (RR: 1.4, 95% CI 1.3–1.5, p<0.001) more likely to comply with hand hygiene practices. 97.5% (359/368) of nursing students felt that the experience would enhance their own hand hygiene practice and would recommend participating in audits as a learning instrument.

**Conclusions:**

With consideration of all stakeholders a sustainable, flexible, programme was implemented. Experiential learning of hand hygiene was a highly valued educational tool and in our project was directly associated with improved hand hygiene compliance. Feedback demonstrated popularity amongst participants and success in achieving its program objectives. While this does not guarantee long term behavioural change it is intuitive that instilling good habits and messages at the early stages of a career will potentially have significant long-term impact.

## Background

The importance of hand hygiene in the prevention of healthcare-associated infections (HCAIs) is not disputed. Hand hygiene is recognized as the single most important element of strategies to prevent HCAIs [1,2]. Essential components for improving hand hygiene compliance rates are monitoring and providing timely performance feedback to health care workers [3-6]. The National University Hospital (NUH) is a 1000 bed tertiary referral hospital in Singapore. In 2008, NUH escalated its hand hygiene efforts with respect to education and institution of standardised auditing. The World Health Organization (WHO) hand hygiene improvement strategy [1,7] was adopted and included hospital-wide direct observations and regular performance feedback to all medical and nursing staff. Results are provided every quarter via emails to all clinical areas, hand hygiene champions, doctors and nursing staff. Results are presented by infection control staff and clinical hand hygiene champions at various hospital platforms including ward-based and hospital administrative meetings and annually at the hand hygiene day hospital-wide event [5]. Throughout 2009 hand hygiene compliance amongst nursing students on clinical attachment to NUH was consistently poor averaging 45% (95% CI; 42%–48%).

Routine audits have been in place since 2008 and capture approximately 800 observations/month hospital-wide. Each discipline and ward receives its own compliance rates via email and these are posted on dedicated display areas in all wards. Results on individuals are not captured. Staff are subject to regular efforts to encourage hand hygiene compliance. This approach did not change throughout the study period. However, given the low compliance rate in nursing students we introduced an intervention to improve hand hygiene compliance for this group.

Our target population was 398 third year nursing students from three nursing schools for pre-registration consolidation placement (PRCP) and transition to practice (TTP) posted to NUH. Although infection control principles including hand hygiene requirements are integrated into the curricula via traditional didactic teaching methods, students still poorly complied with the WHO “My 5 Moments for Hand Hygiene” [8] in the clinical environment. Through experiential learning we aimed to improve the appreciation of the importance of hand hygiene and therefore compliance.

## Methods

A multi-disciplinary team was formed comprising infection control physicians and nurses and the nursing education team. It established an approach to promote better hand hygiene practice among nursing students as well as to drive an innovative learning experience based on previous positive results with medical students [9].

A hand hygiene auditor training programme was incorporated into the orientation programme for final year nursing students as part of their PRCP for diploma students and TTP for degree students.

Audit trainers were infection control nurses who were trained in hand hygiene observation. The training program was based on the WHO “My 5 moments for hand hygiene” approach [1,7,8]. Each training session required one hour and included lectures and practical auditing using the WHO video tools [8]. Students were also trained to document observations from the video by using a standard audit tool. The training video was chosen by the trainer. Students completed the audit form whilst observing all five indications for hand hygiene and the trainer marked the audit form according to the answer sheets. Students were required to achieve 9 out of 10 answers correct (90%) to be deemed certified auditors. If they were unable to achieve 90%, then the trainer provided them with another 10 opportunities. This cycle was repeated until 90%was achieved.

After successful completion of hand hygiene auditor training, students were instructed to observe 20 hand hygiene opportunities of HCWs over 30 minutes during their clinical attachment. Students were required to submit their audit forms and then complete an anonymous 7-question evaluation rating their learning experience using a 4-point Likert scale.

Ethics approval was not sought as direct observation is considered gold standard for hand hygiene audits [5] and have been routinely practiced and accepted at NUH.

### Statistical analysis

Data was aggregated as a monthly average and coded to reflect compliance during the pre-intervention, intervention, and post-intervention period. For visual brevity and comparison with quarterly hospital-wide indicators, student nurses’ compliance was plotted by quarters and two-period moving averages produced. The quarter in which the study intervention occurred was excluded to isolate pre-/post-intervention effects on mean compliance which was expressed as relative risks using the Chi-square test with 95% confidence intervals estimated. Linear mixed model was also used to obtain difference in compliance percentage points with 95% confidence intervals estimated. A p value of <0.05 was taken as significant.

No other interventions occurred concurrently and the Infection Control Link Nurses (ICLNs) located on every ward were unaware of the nursing student efforts.

## Results

Between July and September 2010, the hand hygiene pre-intervention phase commenced and included 398 final year nursing students. Mean compliance of all nursing students in the post-intervention period (October to September 2011) was 64% (95% CI 61%–67%) or 646/1009. These results show a 50% greater likelihood to comply with hand hygiene compliance (RR: 1.5, 95% CI 1.3–1.6, p<0.001) from the pre-intervention mean compliance, 43% (95% CI 39%–47%, 239/555) (Table 1).

**Table 1:** Effect of intervention on hand hygiene compliance in nursing students

	**Opportunities observed**	**Mean compliance**	**Relative risk**	**P value**
	**N**	**(95% CI)**	**(95% CI)**	
**Total**	1564	55 (53–58)		
**Study period**				
Pre-intervention	555	43 (39–47)	1.0	
Post-intervention	1009	64 (61–67)	1.5 (1.3–1.6)	<.001

CI = confidence interval.

**Figure 1:**
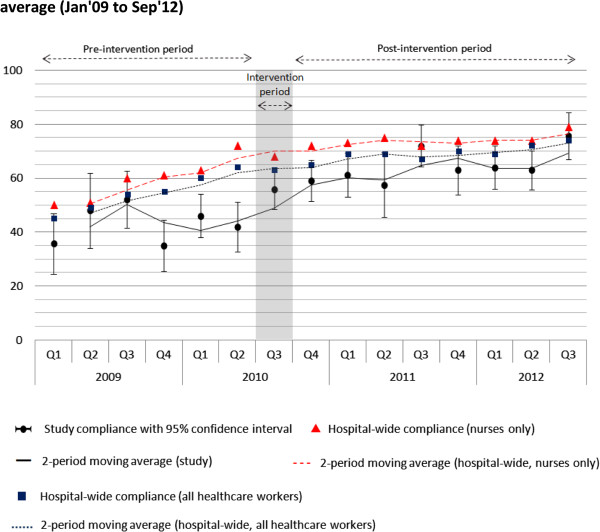
Hand hygiene compliance with 95% confidence intervals and two-period moving average (Jan’09 to Sep’12).

When compared to hospital-wide indicators on nursing staff’s hand hygiene compliance, nursing students in the study registered 16 percentage points less compliance pre-training (see Q1 2009 to Q2 2010 in Figure 1) and decreased to 10 percentage points less compliance post-training. The difference between full-time nursing staff and nursing students was significant but not large p=0.022 (Table 2). Nursing students’ hand hygiene compliance post-intervention was not significantly different to the average hand hygiene compliance of other healthcare workers (p=0.204).

**Table 2:** Linear mixed model demonstrating difference in post-intervention compliance against and hospital-wide indicators

		**Estimate**	**P value**		**Estimate**	**P value**
		**(95% CI)**			**(95% CI)**	
**Pre-intervention**	Hospital-wide (nurses only)	1.0		Hospital-wide (all HCWs)	1.0	
	Study	−16 (−26,–8)	.001	Study	−12 (−20,–3)	.011
**Post-intervention**	Hospital-wide (nurses only)	1.0		Hospital-wide (all HCWs)	1.0	
	Study	−10 (−19,–2)	.022	Study	−5 (−14,3)	.204

CI = confidence interval; HCWs = healthcare workers.

368 (92.5%) students completed the anonymous questionnaire. Almost all students (98%) agreed that “the auditing experience has given me opportunities to observe how infections could be transmitted in the hospital”. The same number agreed that the audit experience had enhanced their hand hygiene practice. More than 94% of students would like to be advocates of hand hygiene. 97% of students recommended more nursing students be involved in hand hygiene audits as a learning instrument.

Students also expressed that:

• “Through the exercise of audit it has given me a greater awareness about hand hygiene in the clinical setting”,

• “Good opportunity for students to better understand importance of hand hygiene and constraints with regards to audit”,

• “It was fun to do the hand hygiene audit and I was more aware of infection control after this audit experience”,

• “It is a good method of experiential learning. We get to put into practice what we have learnt through the audit observations”

## Discussion

Internationally, students have been reported to have low rates of hand hygiene compliance [10]. This was consistent with our baseline data at NUH therefore we considered novel methods to educate our future nurses to improve their hand hygiene practice.

To improve hand hygiene compliance, it is recommended to use multi-modal approaches through a combination of education, workplace reminders and continuous performance feedback [2-6]. Experiential learning is a commonly used (yet still potentially underutilized) learning process in the clinical setting which links education, work and personal development. The distinguishing features of experiential learning includes reflection and transforming of knowledge and meaning that occurs when students reflect on their personal experiences [11]. Individuals have different learning styles and incorporating a range of learning theories, concepts and approaches can serve to build and create an effective learning environment.

Historically, nursing was taught on-the-job. However, within the past decade nursing has entered tertiary institutions and has necessarily reduced student exposure to the clinical setting. Incorporating experiential learning into clinical situations allows students to gain personal practical knowledge previously harvested during pre-tertiary training and closes the increasing theory to practice gap. Nursing staff generally prefer visual or kinaesthetic learning [12] and with the current nursing programs providing less clinical time there is a danger that their learning needs are not being met.

The influence of other HCWs as role models for nursing students should not be underestimated [13]. Allowing nursing students to observe other HCW helps them to understand failures in fundamental practices such as hand hygiene. This understanding may assist nursing students to improve personal practice and disregard poor habits exhibited by senior staff once they commence full-time practice.

It is unlikely that the improvement in hand hygiene compliance by student nurses was due to the Hawthorne effect. Auditors make efforts to be surreptitious and there has been no change in our approach to audits since 2008. However, it is possible that our education initiative made nurse students more aware of the routine auditing practice and that this served as motivation to improve compliance; nevertheless a good result.

Major implementation challenges included coordinating the large volume of students on clinical attachment and the logistical arrangements with various departments for auditor training. Hand hygiene audits are an imperfect measure of the benefits of the programme which is why we attempted validation via an anonymous survey. The intervention is very low risk consuming around 3 hours of a student’s time during their total course and our evidence strongly supports the hypothesis that it improves hand hygiene compliance in this group.

Study limitations include the limited number of audits completed due to the sample size. Furthermore, the exercise was conducted within only one institution although this intervention was able to be adapted to the needs of three nursing schools.

As this is a real-life observation with real-life lessons for the observer, in theory this could be valuable and reproducible in any number of settings internationally and can be sustained with the support of all stakeholders.

## Conclusion

Continued collaboration between nursing schools and clinical training sites is needed to ensure the hand hygiene auditor program remains part of the core content of nursing programs. Essential to the continued success is the support and commitment from all levels of hospital leadership and nursing faculty with recognition of the importance of hand hygiene and its role in reduction of HCAIs.

We introduced a novel educational “hands-on” programme in keeping with modern adult learning techniques. Applying this to a group underperforming in hand hygiene compliance has shown significant benefit. The simplicity of the approach suggests that it could be adapted to other settings irrespective of culture and resources.

Innovative approaches need to be regularly considered to keep the overall hand hygiene program momentum. Future research could involve measurements of these participants’ hand hygiene behavior in years to come to evaluate whether this experiential learning has maintained its benefits in the longer term.

## Competing interests

The authors declare that they have no competing interests.

## Authors’ contributions

SS drafted the manuscript and assisted with the analysis; DF conceived the intervention, oversaw the implementation, and helped to significantly draft the manuscript including analysis; XW participated in the study design and coordination and helped to draft the manuscript; SL assisted with drafting the manuscript; TS performed the statistical analysis and contributed to the manuscript. All authors read and approved the final manuscript.

## References

[B1] Sax H Allegranzi B Chraiti MN Boyce J Larson E Pittet D The World Health Organization hand hygiene observation method Am J Infect Control 2009 37 827 834 10.1016/j.ajic.2009.07.003 20004812

[B2] Pittet D Allegranzi B Sax H Dharan S Pessoa-Silva CL Donaldson L Evidence-based model for hand transmission during patient care and the role of improved practices Lancet Infect Dis 2006 6 641 652 10.1016/S1473-3099(06)70600-4 17008173

[B3] Naikoba S Hayward A The effectiveness of interventions aimed at increasing handwashing in healthcare workers - a systematic review J Hosp Infect 2001 47 173 180 10.1053/jhin.2000.0882 11247676

[B4] Pittet D Hugonnet S Harbarth S Mourouga P Sauvan V Touveneau S Effectiveness of a hospital-wide programme to improve compliance with hand hygiene Lancet 2000 356 1307 1312 10.1016/S0140-6736(00)02814-2 11073019

[B5] WHO Guidelines on Hand Hygiene in Health Care 2009 Geneva: World Health Organization

[B6] Boyce JM Pittet D Guideline for hand hygiene in healthcare settings Recommendations of the Healthcare Infection Control Practices Advisory Committee and the HICPAC/SHEA/APIC/IDSA Hand Hygiene Task Force. Society for Healthcare Epidemiology of America/Association for Professionals in Infection Control/Infectious Diseases Society of America. Morbid Mortal Wkly Rep. Recommendations and reports/Centers for Disease Control 2002 51 1 45 12418624

[B7] Sax H Allegranzi B Uckay I Larson E Boyce J Pittet D ‘My five moments for hand hygiene’: a user-centred design approach to understand, train, monitor and report hand hygiene J Hosp Infect 2007 67 9 21 10.1016/j.jhin.2007.06.004 17719685

[B8] World Health Organization Clean Care is Safer Care - Tools and resources 2009 [updated 2009]. Available from: http://www.who.int/gpsc/5may/tools/en/

[B9] Fisher D Pereira L Ng TM Patlovich K Teo F Hsu LY Teaching hand hygiene to medical students using a hands-on approach J Hosp Infect 2010 76 86 10.1016/j.jhin.2010.04.007 20554349

[B10] Wong TW Tam WW Handwashing practice and the use of personal protective equipment among medical students after the SARS epidemic in Hong Kong Am J Infect Control 2005 33 580 586 10.1016/j.ajic.2005.05.025 16330306 PMC7119109

[B11] Colgrove SR Schlapman N Erpelding C Fuszard B Experiential Learning Innovative Teaching Strategies in Nursing 1995 Gaithersburg: An Aspen Publication

[B12] Frankel A Nurses’ learning styles: promoting better integration of theory into practice Nurs Times 2009 105 24 27 19260265

[B13] Barrett R Randle J Hand hygiene practices: nursing students’ perceptions J Clin Nurs 2008 17 1851 1857 10.1111/j.1365-2702.2007.02215.x 18578759

